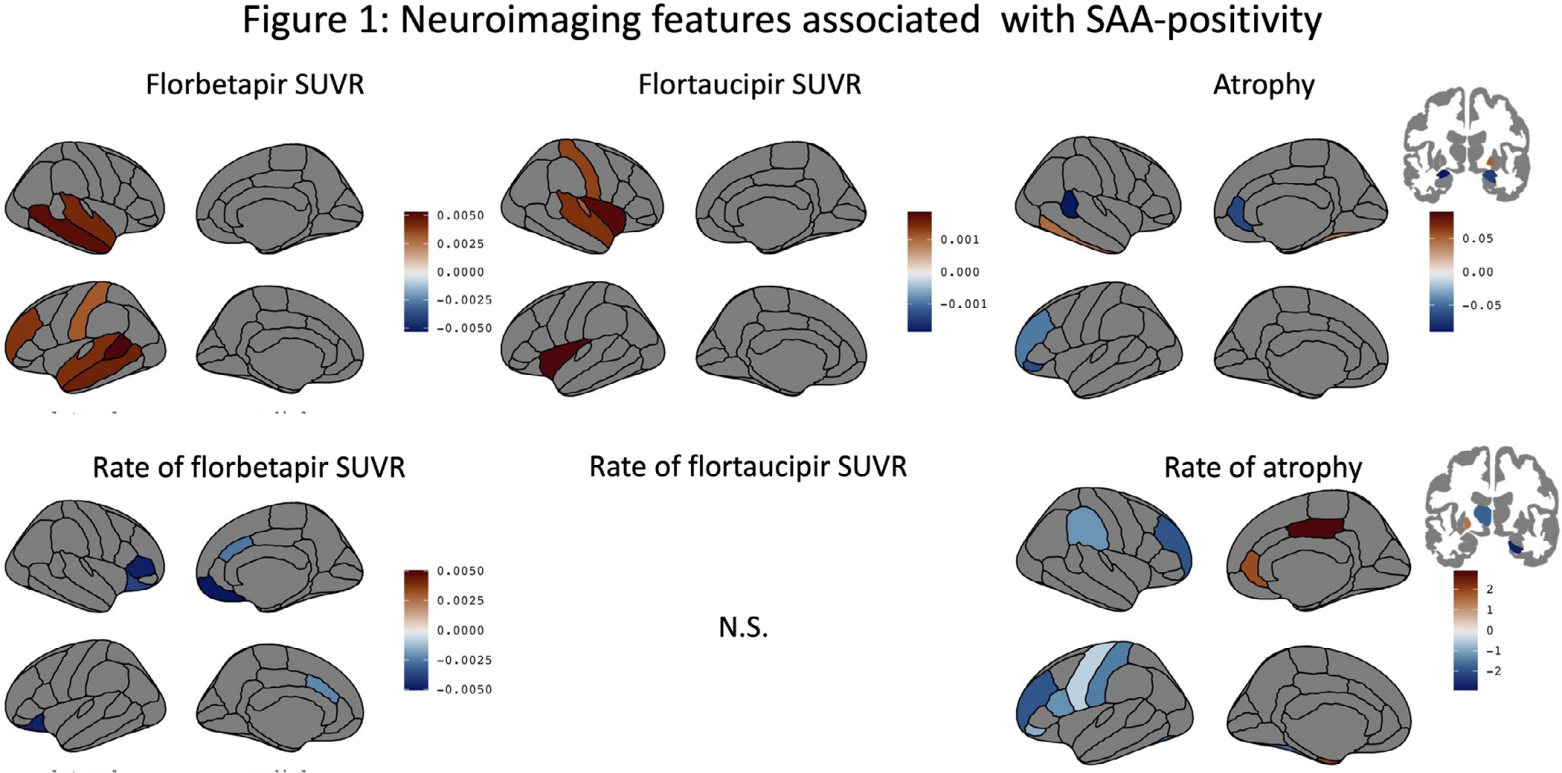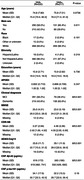# Neuroimaging features of CSF α‐synuclein seed amplification assay positivity in elderly individuals in AD continuum

**DOI:** 10.1002/alz.092637

**Published:** 2025-01-09

**Authors:** Duygu Tosun, Susan M. Landau, Michael S. W. Weiner, Cornelis Blauwendraat, Alzheimer's Disease Neuroimaging Initiative

**Affiliations:** ^1^ University of California, San Francisco, San Francisco, CA USA; ^2^ University of California, Berkeley, Berkeley, CA USA; ^3^ National Institute of Neurological Disorders and Stroke, Bethesda, MD USA

## Abstract

**Background:**

While Aβ and tau pathologies are central to AD, emerging evidence suggests the coexistence of Lewy body pathology (α‐synuclein aggregates), further contributing to neurodegenerative processes. Our objective was to investigate the association between CSF α‐synuclein seed amplification assay (SAA) positivity and in vivo neuroimaging metrics of florbetapir‐PET and flortaucipir‐PET retention, as well as atrophy measured by structural‐MRI, with a focus on Aβ+ cognitively impaired (MCI and Dementia due to AD) individuals.

**Method:**

Participants from ADNI who were Aβ+ (CSF Aβ42<980 pg/mL) and had undergone SAA analysis, along with based on florbetapir‐PET, flortaucipir‐PET, or structural‐MRI scans within one year of CSF sample collection, were included in the analysis (Table 1). Utilizing generalized ridge regression with connectivity‐informed adaptive regularization, SAA‐associated patterns of cross‐sectional neuroimaging features, as well as SAA‐associated patterns of the longitudinal rate of change in neuroimaging features were modeled, adjusting for age, sex, *APOE ε*4 status, and clinical diagnosis with additional adjustments for CSF Aβ42 levels in flortaucipir models and CSF Aβ42 and p‐tau181 levels in atrophy models. Significant associations (95% CI) were determined through 50,000 bootstrap‐based sampling.

**Result:**

In cross‐sectional analysis (Figure 1; top row), compared to SAA‐ individuals, SAA+ was associated with: (1) greater florbetapir‐SUVR, observed in the bilateral middle and superior temporal, banks of superior temporal, left middle frontal and postcentral cortices; (2) elevated flortaucipir‐SUVR in bilateral insula, left superior temporal and postcentral cortices; and (3) greater atrophy in left middle frontal and lateral orbitofrontal, right anterior cingulate and banks of superior temporal cortices, as well as greater GM tissue volume in right inferior temporal cortex. Although the longitudinal sample size was limited, SAA+ was associated with: (1) lower rates of change in florbetapir in bilateral middle/lateral orbitofrontal and anterior cingulate cortices; and (2) higher rates of atrophy in bilateral middle frontal, right inferior parietal, left pre/postcentral and inferior frontal cortices, and lower rates of atrophy in right anterior and posterior cingulate cortices (Figure 1; bottom row).

**Conclusion:**

Our results reveal that SAA‐positivity in individuals on the AD continuum is associated with distinctive patterns of neuroimaging features, highlighting the multifaceted nature of neurodegeneration in AD.